# Clinical Features and Paraclinical Findings in Patients with SARS CoV-2 Pneumonia and the Impact of Pulmonary Rehabilitation on the Instrumental Activities of Daily Living in POST-COVID-19 Patients

**DOI:** 10.3390/jpm13020182

**Published:** 2023-01-20

**Authors:** Paraschiva A. Postolache, Alexandru Nechifor, Ioana Buculei, Ioana Soare, Horia Mocanu, Florin Dumitru Petrariu

**Affiliations:** 1 Internal Medicine Department, Faculty of Medicine, “Grigore T. Popa” University of Medicine and Pharmacy, 700115 Iasi, Romania; 2 Clinical Medical Department, Faculty of Medicine and Pharmacy, “Dunărea de Jos” University, 800008 Galati, Romania; 3 Department of Biomedical Sciences, Faculty of Medical Bioengineering, “Grigore T. Popa” University of Medicine and Pharmacy, 700454 Iasi, Romania; 4 Doctoral School of the Faculty of Medicine, University of Medicine and Pharmacy “Grigore T. Popa”, 700115 Iasi, Romania; 5 Insurance Medicine Department, Faculty of Medicine, “Titu Maiorescu” University, 040441 Bucharest, Romania; 6 Department of Ear, Nose and Throat and Head and Neck, Faculty of Medicine, “Titu Maiorescu” University, 040441 Bucharest, Romania; 7 Department of Preventive Medicine and Interdisciplinarity, “Grigore T. Popa” University of Medicine and Pharmacy, 700115 Iasi, Romania

**Keywords:** SARS CoV-2 pneumonia, IADL, rehabilitation program, respiratory rehabilitation, COVID-19

## Abstract

The functional sequelae grouped under the name “long COVID” most often bring the patient in front of a team of specialists in pulmonary rehabilitation. The aim of this study was to evaluate clinical features and paraclinical findings in patients with SARS CoV-2 (Severe Acute Respiratory Syndrome-Corona Virus-2) pneumonia and to also evaluate the impact of rehabilitation in this category of patients. This study included 106 patients diagnosed with SARS CoV-2. The division of the patients into two groups was performed based on the presence of SAR-CoV-2 pneumonia. Clinical symptoms, biochemical parameters, and pulmonary functional and radiological examinations were recorded and analyzed. The Lawton Instrumental Activities of Daily Living (IADL) scale was applied to all patients. Patients in group I were included in the pulmonary rehabilitation program. Among demographic characteristics, age over 50 years (50.9%; *p* = 0.027) and the female sex (66%; *p* = 0.042) were risk factors for pneumonia in patients with SARS CoV-2. Over 90% of the 26 patients included in the rehabilitation program were less able to feed, bathe, dress, and walk. After 2 weeks, approximately 50% of patients were able to eat, wash, and dress. It is important to provide longer rehabilitation programs in cases of moderate, severe, and very severe COVID-19 patients, in order to significantly improve patients’ participation in daily activities and their quality of life.

## 1. Introduction

Although the Corona Virus Disease of 2019 (COVID-19) pandemic has spread unexpectedly for a long time, reaching a global magnitude since its appearance, there are still unknown things about this condition, and the extent of the sequelae is still under investigation, with up to 19% of cases being severe or critical. It is well known that the condition manifests itself differently in each patient. Over time, a number of risk factors have been described, such as old age, smoking, and the pre-existence of associated diseases. COVID-19 in the elderly was particularly associated with an unfavorable prognosis, a fact supported by the multi-morbidity and fragility of these patients [1,2]. Since the early days of COVID-19, many facets of this disease have been discovered, including skin involvement (urticaria, reddish maculo-papular rashes, and livedo) [1,2,3,4]. 

The functional sequelae grouped under the name “long COVID” are the ones that most often bring the patient in front of a team of specialists in pulmonary rehabilitation [5].

Assessing patients in regard to instrumental activities of daily living (IADL) is a useful and necessary tool that can highlight the fundamental skills needed for independent personal care. It can also provide data on an individual’s functional status; the inability to perform essential activities is associated with a decrease in a patient’s quality of life and shows their dependence on other people or on certain mechanical devices [6,7]. Concerning the main symptoms of “long COVID” syndrome, which persist for a long time after the acute process heals, this tool for assessing the quality of life has proven to be extremely useful in determining the need for rehabilitation and other resources [8]. 

Pulmonary rehabilitation is a process based on the functional evaluation of the patient and consists of the application of appropriate therapies for each patient, therapies aimed at lifestyle changes, educational components, and exercise. These types of programs promote the improvement of the physical and mental status among patients with chronic respiratory diseases. At the same time, long-term adherence to such programs can increase the patient’s effort capacity and quality of life [9,10].

Respiratory rehabilitation is part of the overall treatment of post-COVID-19 patients, alongside risk factor treatment (primarily smoking cessation), the medical treatment of the stable post-COVID-19 patient, and the early prevention and treatment of exacerbations [10]. 

In the COVID-19 pandemic context, virtual outpatient care may be preferable to face-to-face interactions with a physiotherapist following patients who perform rehabilitation exercises for 20 min. This can be easily achieved with the help of a combination of technologies [11].

In hospitalized patients suffering from COVID-19, the aim of respiratory rehabilitation is to improve symptoms of dyspnea, relieve anxiety and depression, reduce complications, prevent and improve lung dysfunction, reduce disability, preserve function to the maximum extent, and improve the quality of life [12].

Given the functional limitations imposed by residual symptoms after Severe Acute Respiratory Syndrome—Corona Virus-2 (SARS CoV-2) infection, as well as the predominant impairment of the comorbid elderly (whether or not already in the process of pulmonary rehabilitation for other conditions), the aim of this study was to evaluate the relationship existing between IADL and the post-COVID status of the patient, as well as to determine the exact moment when pulmonary rehabilitation programs are required to begin, and also to evaluate the impact of rehabilitation on these patients.

## 2. Materials and Methods

### 2.1. Study Design and Setting 

A retrospective observational analytical study was conducted on 106 patients diagnosed with SARS CoV-2, consecutively admitted to the Respiratory Rehabilitation Clinic of the Rehabilitation Clinical Hospital in Iasi, Romania, from August 2020 to August 2021, for an inpatient pulmonary rehabilitation program. 

### 2.2. Patient Selection

The study group consisted of 106 patients diagnosed with SARS CoV-2 infection. Informed consent was obtained from every participant in the study. Data were extracted in the last part of September 2021 from the medical records, ensuring the patient’s confidentiality, in accordance with the current regulations. The survey was conducted according to the guidelines of the Helsinki Declaration and approved by the Ethics Committee of the Rehabilitation Clinical Hospital in Iasi, Romania, on 13 September 2021, number 23650/23651.

A confirmed diagnosis of SARS CoV-2 infection via a reverse transcription polymerase chain reaction (RT-PCR) assay was the main inclusion criterion in the study. The division of the patients in into two groups was performed based on the presence of SAR-CoV-2 pneumonia: Group I—26 patients with pneumonia and group II—80 patients with a definite diagnosis of non-pneumonia. The patient inclusion in the two study groups was performed according to the clinical symptoms at hospital admission (such as dyspnea, chest pain, dry cough, fever, and myalgia) and the imaging results obtained during hospital admission.

### 2.3. Respiratory Rehabilitation

In order to assess the impact of pulmonary rehabilitation on the exercise capacity of this group, these patients benefited from a multidisciplinary team intervention, consisting of a pulmonologist, functional exploration physician, physiotherapist, psychologist, and nutritionist. The entire team provided educational support and advice and psychological, nutritional, and behavioral counseling.

All patients received brochures and information about the importance of these programs in order to increase compliance with these programs. Regarding the exercise sessions, aerobic exercises adapted to the patient’s functional status were performed, such as walking, brisk walking, and slow running, all of which were performed on the treadmill or cycle ergometer (from a diminished intensity and duration to a progressive increase). At the same time, breathing exercises were performed in order to adjust the chest posture and the breathing rhythm, in addition to diaphragmatic training, stretching exercises, and cough exercises [13]. The short duration of the rehabilitation programs in the hospital required their continuation at home.

Two strategies were employed: The first one is based on consolidated principles of early respiratory rehabilitation, including mobilization and psychological support, that has to be started during the acute phase of illness; the second strategy is based on the Chinese and Italian experiences, countries that had to face the severe forms of COVID-19 pathology early during the pandemic, experiencing a crisis in rehabilitation services [12,13,14,15].

#### Rehabilitation Evaluation

Clinical evaluation: Physical examination, imaging, laboratory, lung function, and insurance medicine evaluation (according to International Classification of Functionality and Disability, ICF criteria—Structures, Functions, Participation, Activities) were performed.

For the evaluation of exercise and respiratory function, respiratory muscle strength was assessed using maximum inspiratory pressure/maximum expiratory pressure; for muscle strength, isokinetic muscle testing was used; the 6-min walk test was performed in order to assess aerobic exercise capacity; IADL was used for the assessment of daily living ability; and the joint range of motion measurement was also used. 

All patients completed the Lawton IADL scale [16], which contains eight items, measuring eight parameters, rated from 0 (low functioning) to 8 (high functioning), and was accompanied by a written questionnaire. The Lawton IADL scale was developed in order to assess the activities used for functioning in community settings, named IADL, unlike basic activities, such as eating, bathing, and toileting, named “activities of daily living” (ADL). The scale covers areas such as shopping, transportation, managing finances, house cleaning and laundry, managing communication with other people, administering medication, and food preparation. While the patients completed the questionnaire (10–15 min), they received explanations and support from the medical staff. 

Moreover, the severity of resting dyspnea was assessed with the modified Medical Research Council (mMRC) scale, and the severity of effort dyspnea was evaluated with the help of the Borg scale, scales that vary from 0–5 and 0–10, respectively [16]. 

According to the current recommendations, the group that followed a pulmonary rehabilitation program consisted of 26 patients diagnosed with pneumonia. The duration of this program was 2 weeks, with 5 sessions per week and each session lasting 30 min [9,10]. 

All participants completed the IADL scale before starting the rehabilitation program, as well as at the end of the 2-week rehabilitation program.

### 2.4. Data Collection and Management

Electronic medical records were retrospectively screened, and relevant data were independently extracted for all the participants included in the study. The relevant data extracted were included in de-identified form in an excel spreadsheet. All data were stored in password-protected electronic documents with access only for the authors of the study.

Patient demographic data and baseline clinical characteristics including age, gender, and comorbidities were recorded. Clinical data consisted of symptoms and smoking status. Paraclinical data consisted of lung function test results, blood testing laboratory parameters, and radiological examinations.

The data collected also included information concerning the therapeutic approach of patients.

### 2.5. Statistical Analysis

Data were systematized and centralized in an SPSS 18.0 database and processed using the appropriate statistical functions. A 95% confidence interval was used in the data presented. Primary indicators (minimum, maximum, and frequency), mean value indicators (mean, median), and dispersion indicators (standard deviation (SD), standard error (SE), and confidence interval (CI) for the mean) were used for descriptive statistical analysis. The Skewness test (−2 < Sk < 2) was used to validate the normality of the value series for continuous variables.

Qualitative significance tests, such as the Chi2 test, were used to compare the distributions of frequencies. The odds ratio (OR) and relative risk (RR) were used to measure the association between exposure and outcome. The t-Student test was used to compare the means of any two normally distributed variables. The plotting of the receiver operator characteristic (ROC) curve, which defines the area under the curve (AUC) and where the false-positive rate (1-specificity) is placed on the abscissa and the true-positive rate (sensitivity) on the ordinate, allowed the analysis of the sensitivity/specificity balance.

## 3. Results

In this retrospective observational analytical study, 106 patients diagnosed with SARS COV-2 infection were included; clinical features and biological variables were statistically assessed. Table 1 shows the descriptive statistics of demographical data, signs, and symptoms of the patients included in the study. 

The median age of the patients was 50 years old. A predominance of the female gender was observed in all groups; in group I, 50% of the participants were female, and in group II, 71.3% were female (Table 1). A dry cough was the most predominant symptom in both groups (42.3% and 13.8%). Fever was the second most prevalent symptom in group I (30.8%) followed by chest pain (23.1%), myalgia (23.1%), anosmia (19.2%), and dyspnea (19.2%). In group II, the second most prevalent symptom was a productive cough and anosmia (10.0%), followed by ageusia (8.8%) and chest pain (7.5%). 

Among demographic characteristics, age over 50 years was a risk factor for pneumonia in patients with SARS CoV-2 (*p* = 0.027). The female gender (*p* = 0.042) was significantly associated with pneumonia in SARS CoV-2 patients (Table 1).

The percentage of cases with dyspnea was 4.81 (3.29–7.04) times higher in patients with SARS CoV-2 and pneumonia (19.2% vs. 0 %; *p* = 0.001). Fever was significantly identified in patients with SARS CoV-2 and pneumonia (30.8 % vs. 3.8 %; *p* = 0.001), with the estimated risk being 3.84 (2.21–6.66) times higher. Dry cough was significantly more common in patients with SARS CoV-2 who also associated pneumonia (42.3 % vs. 13.8 %; *p* = 0.003), the estimated risk being 2.80 (1.51–5.21) times higher. In SARS CoV-2 patients, with an estimated risk of more than 4 (2.98-5.91) or higher, paresthesia was more frequently associated with pneumonia (3.8 % vs. 0 %; *p* = 0.092), but the low number of cases does not allow extrapolation of the results (Table 1).

A multivariate analysis was conducted, and Table 2 shows the impact of demographical data on pneumonia. Gender and age may be confounding factors in the determination of pneumonia (*p* = 0.014).

Table 3 shows the sensitivity/specificity balance of signs and symptoms in the determination of pneumonia in patients with SARS CoV-2.

**Figure 1:**
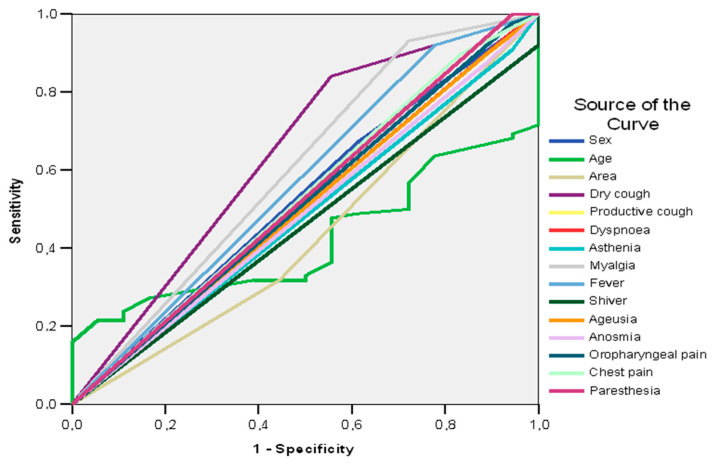
AUC. The sensitivity/specificity balance of signs and symptoms in the determination of pneumonia in patients with SARS CoV-2.

In the case study, the ROC curve shows that, among the signs and symptoms of patients with SARS CoV-2, only dry cough (AUC = 0.643; 95%CI: 0.512–0.774; *p* = 0.029) and fever (AUC = 0.635; 95%CI: 0.500–0.770; *p* = 0.039) can be good predictors of pneumonia (AUC > 0.600) (Table 2, Figure 1).

In Table 4, risk factors associated with SARS CoV-2 pneumonia patients according to personal pathological history can be observed.

Among the personal pathological history, only cancers induced a relatively higher risk of pneumonia in patients with SARS CoV-2 [RR or RT = 4.81 (3.29–7.04); *p* = 0.001] (Table 3).

Table 5 shows the mean values of laboratory parameters of the patients included in the study.

In patients with SARS CoV-2 and pneumonia, the mean values of some hematological and biochemical laboratory parameters showed significantly higher differences: White blood cells (6.22 vs. 4.80; *p* = 0.039), polymorphonuclear cells (PMN) (64.18% vs. 51.39%; *p* = 0.007), C-reactive protein (CRP) (4.86 mg/dL vs. 0.78 mg/dL; *p* = 0.001), erythrocyte sedimentation rate (ESR) (22.65 mm/h vs. 11.36 mm/h; *p* = 0.006), fibrinogen (446.31 mg/dL vs. 216.91 mg/dL; *p* = 0.001), ferritin (283.90 kD vs. 71.54 kD; *p* = 0.001), creatinine (1.16 mg/dL vs. 0.74 mg/dL; *p* = 0.006), glycemia (106.71 mg/dL vs. 86.53 mg/dL; *p* = 0.05), alanine aminotransferase (ALT) (39.63 UI/L vs. 26.48 UI/L; *p* = 0.05), aspartate aminotransferase (AST) (32.01 UI/L vs. 19.58 UI/L; *p* = 0.001), gamma-glutamyl transferase (GGT) (50.63 UI/L vs. 21.57 UI/L; *p* = 0.001), and bilirubin (0.51 mg/dL vs. 0.30 mg/dL; *p* = 0.001) (Table 5).

**Figure 2:**
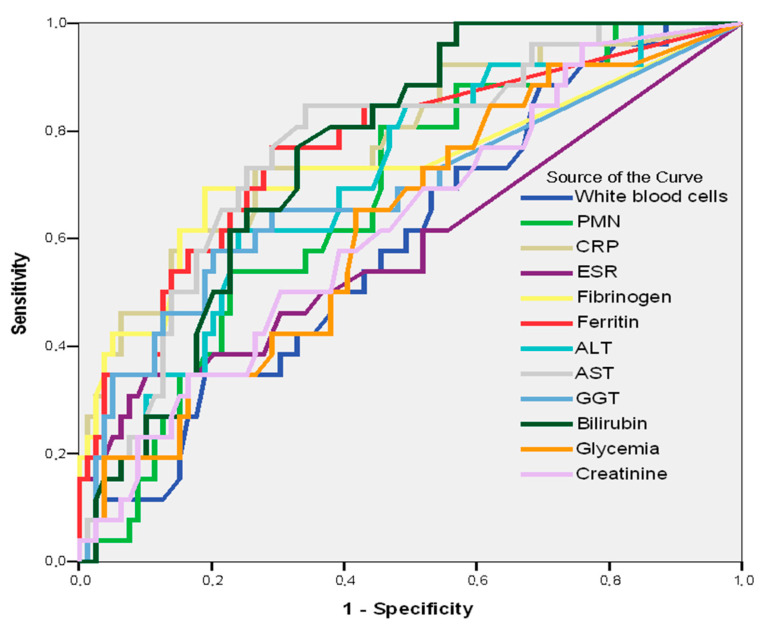
AUC. Laboratory parameter predictors of pneumonia in patients with SARS CoV-2.

In Table 6 and Figure 2, the sensibility/specificity balance of laboratory parameters in predicting pneumonia in patients with SARS CoV-2 can be observed.

By tracing the AUC, it is revealed that some hematological constants—CRP (AUC = 0.779; 95%CI: 0.672–0.886; *p* = 0.001), AST (AUC = 0.769; 95%CI: 0.668–0.871; *p* = 0.001), ferritin (AUC = 0.767; 95%CI: 0.657–0.877; *p* = 0.001), bilirubin (AUC = 0.758; 95%CI: 0.665–0.851; *p* = 0.001), fibrinogen (AUC = 0.734; 95%CI: 0.605–0.863; *p* = 0.001), and ALT (AUC = 0.706; 95%CI: 0.595-0.817; *p* = 0.002)—may be good predictors of pneumonia through SARS CoV-2 (Table 6, Figure 2).

In this study, there are no significant differences between systolic blood pressure, diastolic blood pressure, heart rate, and oxygen saturation (SpO2) in patients with or without pneumonia and SARS CoV-2 (Table 7).

Antibiotics (76.9%; *p* = 0.001), including Clarithromycin (19.2%; *p* = 0.017), ceftriaxone (53.8%; *p* = 0.001), Kaletra (lopinavir 80 mg and ritonavir 20 mg) (46.2%; *p* = 0.001), and Dexamethasone (57.7%; *p* = 0.001), were used predominantly in the treatment of patients with SARS CoV-2 and pneumonia. Paracetamol was frequently used in both study groups (80.8% vs. 78.7%; *p* = 0.824) (Table 8).

**Figure 3:**
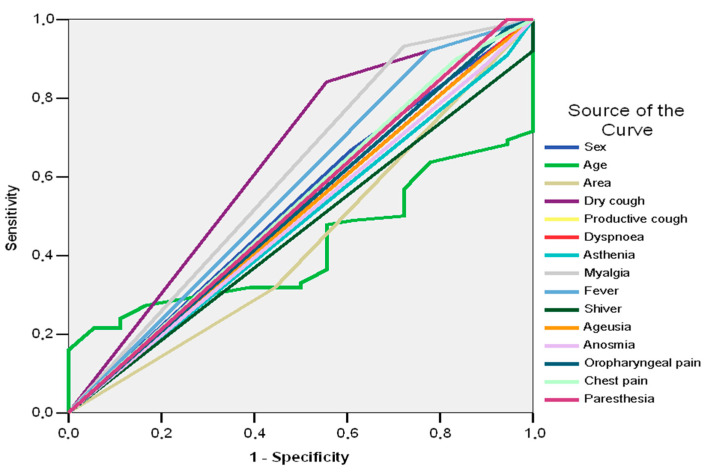
AUC. The sensitivity/specificity balance of demographical data, signs, and symptoms in the determination of unfavorable evolution in patients with SARS CoV-2 and pneumonia.

Good predictors of adverse outcome in patients with SARS CoV-2 and pneumonia prove to be dry cough (AUC = 0.643; 95%CI: 0.491–0.795; *p* = 0.057) and myalgia (AUC = 0.605; 95%CI: 0.448–0.761; *p* = 0.162) (Table 9, Figure 3).

The results in Table 3 and Table 9 show that dry cough is a good predictor of pneumonia (AUC = 0.643; 95%CI: 0.512–0.774; *p* = 0.029) and unfavorable evolution (AUC = 0.643; 95%CI: 0.491–0.795; *p* = 0.057). Myalgia was a good predictor of unfavorable evolution (AUC = 0.605; 95%CI: 0.448–0.761; *p* = 0.162), but not of pneumonia (AUC = 0.584; 95%CI: 0.450–0.718; *p* = 0.199). Fever was a good predictor of pneumonia (AUC = 0.635; 95%CI: 0.500–0.770; *p* = 0.039), but not for the unfavorable evolution, and a good predictor of pneumonia (AUC = 0.571; 95%CI: 0.416–0.726; *p* =0.342).

All 26 participants included in group I were evaluated by the Lawton IADL scale, and dyspnea was also assessed (using the mMRC and Borg scale) both before the start of the pulmonary rehabilitation program and at the end of the two weeks. Results can be seen in Table 10, showing the IADL evaluation in SARS CoV-2 patients with pneumonia before and after the rehabilitation program.

By analyzing the participants’ answers to the eight-item questionnaire, we found that most participants revealed a final score of reduced functionality. Over 90% of the 26 patients included in the rehabilitation program were less able to feed, bathe, dress, walk, and cook. After 2 weeks, approximately 50% of patients were able to eat, wash, and dress. Walking improved in 30% of patients (Table 9).

## 4. Discussion

Although the benefit of pulmonary rehabilitation programs has been demonstrated in multiple studies, this study is among the first to analyze the impact of pulmonary rehabilitation programs on IADL in post-COVID-19 patients. As a new condition, the long-term consequences are not yet known, but this has not been an obstacle in including these patients in rehabilitation programs. It would be even more beneficial to each patient if this program could be multidisciplinary or cross-domain, covering multiple specialties and extending far beyond hospital care to community rehabilitation services as well, with the inclusion of tertiary, secondary, and/or primary-care facilities/levels. Some experts recommend, according to COVID-19’s clinical signs and symptoms, that the patient’s rehabilitation has to include pulmonary, physical, and psychosocial rehabilitation, as in chronic obstructive pulmonary disease (COPD) [17,18,19,20].

Pulmonary rehabilitation has many goals, such as maintaining flow through the respiratory tract, improving ventilation, decreasing the patient’s dyspnea, improving respiratory muscle function and chest mobility, reducing the respiratory rate, improving overall endurance, achieving relaxation with reduced or abolished anxiety and/or depression, and an overall improved quality of life for the patient [21,22].

During the first wave in Italy, almost one out of seven physical therapists tested positive on the COVID-19 test. Considering personal- and work-related exposures, healthcare organizations should adopt preventive measures and adequate preparation in order to prevent high rates of infection during future pandemics [15].

Previously published studies [23,24] have identified the main symptoms that patients face even after 6 months after curing the infection: Dyspnea, dry cough, myalgia, fatigue, arthralgia, and insomnia. Similarly, the 106 participants reported a range of symptoms such as dry cough, dyspnea, asthenia, myalgia, anosmia and ageusia, and chest pain. Among the participants with COVID-19-induced pneumonia, we found that most of them were women over 50 years of age. Moreover, the presence of cancer induced an approximately 5-fold increased risk of pneumonia in patients with SARS CoV-2.

By evaluating the hematological and biochemical parameters, we found an association between certain biological criteria and the prediction of pneumonia risk. Thus, elevated values of CRP, ferritin, bilirubin, fibrinogen, AST, and ALT can be good predictors of pneumonia in patients with SARS CoV-2. Regarding the therapeutic strategy applied to the participants in the study group, by analyzing the data recorded in the observation sheets, we found that the most common treatment consisted of antibiotics, Dexamethasone (a powerful synthetic glucocorticoid with anti-inflammatory effects) [25], and Kaletra (lopinavir 80 mg and ritonavir 20 mg) (an antiretroviral drug also used in HIV patients) [26].

Previously published studies [27,28] aimed at determining the negative prognostic factors in patients with COVID-19; they presented dry cough, fatigue, sputum, chest pain, myalgia, and arthralgia as elements with significant prognostic value on the quality of life. Concerning the patients included in the study, the presence of dry cough and myalgia (in those with SARS CoV-2 and pneumonia) led to an unfavorable prognosis. 

A substantial number of people are on sick leave due to COVID-19. Sick leave may be protracted, and sick leave for long COVID is rather common. The severity of COVID-19 (needing inpatient care), prior sick leave, and age all seem to predict the likelihood of longer sick leave [29]. In addition to socio-demographic risk factors, sick-leave diagnoses constitute an important medium and long-term predictor of disability pension among men and women on long-term sickness absence [30].

Individuals discharged after a severe course of COVID-19 frequently present with persisting physical and cognitive dysfunctions after hospital discharge. Those patients significantly benefit from multi-disciplinary inpatient rehabilitation [31]. The World Health Organization (WHO) reported that a patient’s respiratory system is affected significantly even in the mild stages of pneumonia, with more extensive damage in severe cases or in the ARDS stage of the disease. Even now, there is not sufficient data on the extent to which this disease affects the body [18].

Spielmanns et al. reported in their study from 2021 that up to one-third of COVID-19 patients registered severe respiratory complications or even ARDS, with an associated physical impairment and lung function. Their findings revealed that post-COVID-19 patients had a better recovery as compared to patients who suffered from COVID-19, but who also had previous chronic diseases (especially pulmonary ones) [32]. 

Another study from 2021, by Chikhanie et al., reported that patients suffering from COVID-19 who were hospitalized for a long time had severe short-term impairment consisting of muscle function impairment with a limited exercise capacity, causing a negative impact overall on the patient’s quality of life [33]. Surely, patients with such pulmonary function impairment benefit from rehabilitation programs, but to what extent? The same study revealed that the faster the pulmonary recovery starts (post-ICU admittance) and the longer it lasts, the better results are yielded, thus, patients have an overall improved physical capacity, balance, muscle strength, and psycho-social status [33]. 

Rehabilitation measures need to be taken at least in the acute phase of recovery, due to the fact that even patients without prior diseases registered for weeks ongoing symptoms such as chest pain and/or fatigue. The rehabilitation programs need to be developed by focusing on the new COVID-19 era and ensuring that both the rehabilitation itself is successful and that the possibility of viral spread is at a minimum [18,34]. 

Pulmonary rehabilitation has already proven its beneficial effects, long before COVID-19 started, in patients suffering from chronic lung diseases such as COPD. The already set respiratory rehabilitation programs can be used for patients who are in the post-acute phase of the disease, recovering, but presenting with physical impairment and/or symptoms. Exercise training is the main component in lung rehabilitation, and it includes aerobics and/or resistance exercises; they are already known for having beneficial effects on the patient’s physical function, which is usually affected during long periods of hospital admission or by having a sedentary lifestyle. Respiratory rehabilitation exercises also have a positive impact on muscle strength, exercise capacity, and the patient’s overall quality of life [35,36].

Considering the easy way that the SARS CoV-2 virus (responsible for COVID-19) spreads, could pulmonary rehabilitation benefit from alternatives to in-person models? There is new evidence that supports the so-called online telerehabilitation, supervised by a specialist in this field. A group of Canadian researchers developed a standard pulmonary rehabilitation program (inspired by a COPD program), which can be self-managed and delivered either personally or via web platforms [37,38,39]. Telerehabilitation, part of telehealth [40], can be delivered electronically or to a nearby hospital facility, and is the solution for hospital-based, classical rehabilitation programs, which provide long-distance rehabilitation services. What is convenient and practical is that there are many ways in which it can be provided to the patient: Virtual check-ins, asynchronous e-visits, real-time, a two-way visit with audio, video, or both, long-distance evaluations of images and/or videos, or even patient management and assessment via the telephone. Although it was first mentioned in 1998 [38,39], telerehabilitation needs further study in order to really determine the beneficial effects it can have on a patient’s rehabilitation, especially in comorbid COVID patients.

Results in Table 10 show that at the beginning of the pulmonary rehabilitation program, over 90% of the 26 patients were less able to feed, bathe, dress, walk, and cook. After 2 weeks, approximately 50% of patients became able to eat, wash, and dress. Walking improved in 30% of patients which shows the importance of pulmonary rehabilitation in increasing the QOL of patients. Little to no impact was seen on the ability to cook and manage finances, and a possible explanation is that cooking and managing finances are more physically and mentally complex tasks [41]. Studies conducted in the field of cognitive and psychological performance show that patients with COVID-19 suffer from mild cognitive impairment, particularly in the areas of language and memory. Moreover, problems with word-finding and short-term memory were reported [41]. In a study conducted on patients’ recovery from COVID-19, Jaywant et al. showed that working memory was impaired in 55% of the study participants, speed of processing was impaired in 40%, and divided attention was impaired in 46% [42]. This study has several limitations, one of which is represented by the small number of participants. We studied only the acute effects of the interventions on the activities of daily living, and thus, long-term effects could not be evaluated. A limitation of this study is the impact on the quality of life (QOL) of patients with COVID-19, regardless of the time of discharge after hospitalization and recovery. Healthcare providers need to focus on assessing patients’ needs and implementing effective strategies to quantify the quality of life of post-COVID-19 patients who have or have not had pulmonary rehabilitation. More studies are needed to investigate the impact that pulmonary rehabilitation has not only on the activities of daily living but also on the clinical manifestation of the disease, effort capacity, muscle strength, and lung capacity.

The authors of this study consider that the implementation of pulmonary rehabilitation programs in the treatment of this category of patients can be considered an interesting therapeutic tool that needs to be used after a thorough assessment of the abilities, needs, and comorbidities of each patient. Especially in countries with a high prevalence of SARS CoV-2 infections and a subsequent number of patients with a temporary pulmonary disability, pulmonary rehabilitation programs may represent a cost-efficient option, able to improve the quality of life of patients. Moreover, the findings of the present study could be exploited by researchers and clinicians in order to better understand and address the impairments and rehabilitation needs of COVID-19 patients.

## 5. Conclusions

As it is revealed by the results presented in this study, we can conclude that pulmonary rehabilitation programs have significant benefits for these patients; the symptoms with which they presented in our clinic were visibly improved after rehabilitation. Given the data presented, we believe that lung rehabilitation programs should be included in the management recommendations of patients cured of COVID-19, thus ensuring optimal social and functional reintegration, but also preventing the functional demise that can be overlooked due to its slow evolution.

After the rehabilitation program, eating, bathing, dressing, and walking improved. It is important to provide longer pulmonary rehabilitation programs (beyond 8 weeks) in moderate, severe, and very severe respiratory disease of SARS CoV-2 patients, so that their participation in daily activities and their quality of life can be improved.

## Figures and Tables

**Table 1:** Descriptive statistics of demographical data, signs, and symptoms of the patients included in the study.

Comorbidities	Total n = 106	SARS CoV-2 and Pneumonia n = 26	SARS CoV-2 and Non-Pneumonia n = 80	Chi^2^ Test *p*	Estimate Risk (95%CI)
**Demographic Data**					
Age (years; median/interval)	50/3-90	57/23-86	48/3-90	0.009	-
Female	70 (66.0%)	13 (50.0%)	57 (71.3%)	0.042	0.70 (0.47–1.06)
≥50 years	54 (50.9%)	18 (69.2%)	36 (45.0%)	0.027	1.94 (1.01–3.74)
Urban	70 (66.0%)	17 (65.4%)	53 (66.3%)	0.557	0.97 (0.48–1.96)
Smoking	23 (21.7%)	5 (19.2%)	18 (21.7%)	0.727	0.85 (0.35–2.07)
**Clinical Findings**					
Dry cough	22 (20.8%)	11 (42.3%)	11 (13.8%)	0.003	2.80 (1.51–5.21)
Productive cough	12 (11.3%)	4 (15.4%)	8 (10.0%)	0.465	1.42 (0.59–3.43)
Dyspnoea	5 (4.7%)	5 (19.2%)	0 (0.0%)	0.001	4.81 (3.29–7.04)
Asthenia	9 (8.5%)	3 (11.5%)	6 (7.5%)	0.534	1.41 (0.52–3.79)
Myalgia	11 (10.4%)	6 (23.1%)	5 (6.3%)	0.023	2.59 (1.33–5.04)
Fever	11 (10.4%)	8 (30.8%)	3 (3.8%)	0.001	3.84 (2.21–6.66)
Shiver	7 (6.6%)	3 (11.5%)	4 (5.0%)	0.271	1.85 (0.73–4.66)
Ageusia	11 (10.4%)	4 (15.4%)	7 (8.8%)	0.354	1.57 (0.66–3.72)
Anosmia	13 (12.3%)	5 (19.2%)	8 (10.0%)	0.232	1.70 (0.78–3.73)
Oropharyngeal pain	3 (2.8%)	1 (3.8%)	2 (2.5%)	0.728	1.37 (0.27–7.05)
Chest pain	12 (11.3%)	6 (23.1%)	6 (7.5%)	0.041	2.35 (1.18–4.67)
Paraesthesia	1 (0.9%)	1 (3.8%)	0 (0.0%)	0.092	4.20 (2.98–5.91)

Abbreviations: SARS CoV-2, Severe Acute Respiratory Syndrome-Corona Virus-2; CI, confidence interval.

**Table 2:** Multivariate analysis. Impact of demographical data on pneumonia.

Model	R	R Square	Adjusted R Square	Std. Error of the Estimate	Change Statistics				
					R Square Change	F Change	df1	df2	Sig. F Change
1	0.193 ^a^	0.037	0.028	0.426	0.037	40.026	1	104	0.047
2	0.304 ^b^	0.093	0.075	0.416	0.055	60.290	1	103	0.014
3	0.305 ^c^	0.093	0.066	0.418	0.000	0.030	1	102	0.862
4	0.332 ^d^	0.110	0.075	0.416	0.017	10.947	1	101	0.166

^a^ Predictors: (Constant), Sex; ^b^ Predictors: (Constant), Sex, Age; ^c^ Predictors: (Constant), Sex, Age, Area; d Predictors: (Constant), Sex, Age, Area, Smoker.

**Table 3:** The sensitivity/specificity balance of signs and symptoms in the determination of pneumonia in patients with SARS CoV-2.

Sign	AUC	Standard Error	*p*	95%CI	
				−95%CI	+95%CI
Dry cough	0.643	0.067	0.029	0.512	0.774
Productive cough	0.527	0.067	0.681	0.396	0.658
Dyspnoea	0.596	0.069	0.142	0.460	0.732
Asthenia	0.520	0.066	0.758	0.390	0.651
Myalgia	0.584	0.068	0.199	0.450	0.718
Fever	0.635	0.069	0.039	0.500	0.770
Shiver	0.533	0.067	0.618	0.401	0.664
Ageusia	0.533	0.067	0.612	0.402	0.664
Anosmia	0.546	0.067	0.481	0.414	0.678
Oropharyngeal pain	0.507	0.066	0.918	0.378	0.636
Chest pain	0.578	0.068	0.234	0.444	0.712
Paraesthesia	0.519	0.067	0.769	0.389	0.650

Notes: The test result variable(s): Dry cough, Productive cough, Dyspnoea, Asthenia, Myalgia, Fever, Shiver, Ageusia, Anosmia, Oropharyngeal pain, Chest pain, and Paresthesia have at least one tie between the positive actual state group and the negative actual state group. Statistics may be biased. a. Under the nonparametric assumption b. Null hypothesis: True area = 0.5; Abbreviations: AUC, area under the curve; CI, confidence interval.

**Table 4:** Risk factors in SARS CoV-2 patients with pneumonia according to personal pathological history.

Comorbidities	Total n = 106	SARS CoV-2 and Pneumonia n = 26	SARS CoV-2 and Non-Pneumonia n = 80	Chi^2^ Test *p*	Estimate Risk (95%CI)
HBP	53 (50.0%)	13 (50.0%)	40 (50.0%)	1.000	1.00 (0.51–1.95)
Diabetes mellitus	8 (7.5%)	3 (11.5%)	5 (6.3%)	0.396	1.60 (0.61–4.19)
Obesity	16 (15.1%)	2 (7.7%)	14 (17.5%)	0.197	0.47 (0.12–1.79)
Cancers	5 (4.7%)	5 (19.2%)	0 (0.0%)	0.001	4.81 (3.29–7.04)
Autoimmune disease	5 (4.7%)	0 (0.0%)	5 (6.3%)	0.089	1.35 (1.20–1.51)
COPD	7 (6.6%)	2 (7.7%)	5 (6.3%)	0.800	1.18 (0.35–4.00)
Ischemic heart disease	17 (16.0%)	6 (23.1%)	11 (13.7%)	0.276	1.57 (0.74–3.33)
Psychiatric illness	4 (3.8%)	1 (3.8%)	3 (3.8%)	0.982	1.02 (0.18–5.76)
Neurological disease	9 (8.5%)	2 (7.7%)	7 (8.8%)	0.865	0.90 (0.25–3.20)
Digestive disease	13 (12.3%)	5 (19.2%)	8 (10.0%)	0.232	1.70 (0.78–3.73)
Hematological disease	5 (4.7%)	1 (3.8%)	4 (5.0%)	0.805	0.81 (0.14–4.82)

Abbreviations: SARS CoV-2, Severe Acute Respiratory Syndrome-Corona Virus-2; CI, confidence interval; HBP, arterial hypertension; COPD, chronic obstructive pulmonary disease.

**Table 5:** Mean values of laboratory parameters in blood of the patients included in the study.

Paraclinical Parameters	Total n = 106	SARS CoV-2 and Pneumonia n = 26	SARS CoV-2 and Non-Pneumonia n = 80	t-Student Test *p*
White blood cells	5.15 ± 3.08	6.22 ±4.05	4.80 ± 2.63	0.039
PMN%	54.53 ± 21.16	64.18 ± 9.89	51.39 ± 22.88	0.007
PMN-abs	2.89 ± 2.02	3.55 ± 1.89	2.68 ± 2.03	0.050
Lymphocytes	25.29 ± 12.02	24.82 ± 8.46	25.45 ± 13.01	0.819
Lymphocytes -abs	1.23 ± 0.71	1.27 ± 0.48	1.22 ± 0.77	0.747
CRP	1.79 ±0.40	4.86 ± 1.45	0.78 ± 1.30	0.001
ESR	14.13 ± 1.80	22.65 ± 5.36	11.36 ± 1.54	0.006
Fibrinogen	273.18 ± 25.82	446.31 ± 60.22	216.91 ± 25.26	0.001
Ferritin	123.63 ± 19.63	283.90 ± 61.73	71.54 ± 12.11	0.001
Urea	23.23 ± 15.44	26.42 ± 16.20	22.19 ± 15.14	0.227
Creatinine	0.85 ± 0.71	1.16 ± 1.18	0.74 ± 0.43	0.006
Glycemia	91.48 ± 47.52	106.71 ± 51.66	86.53 ± 45.34	0.050
ALT	29.74 ± 31.08	39.63 ± 27.47	26.48 ± 31.66	0.050
AST	22.63 ± 13.66	32.01 ± 13.89	19.58 ± 12.19	0.001
GGT	28.70 ± 4.80	50.63 ± 12.07	21.57 ± 4.79	0.001
Bilirubin	0.35 ± 0.27	0.51 ± 0.19	0.30 ± 0.28	0.001

Abbreviations: SARS CoV-2, Severe Acute Respiratory Syndrome-Corona Virus-2; PMN, polymorphonuclear cells; CRP, C reactive protein; ESR, erythrocyte sedimentation rate; AST, aspartate aminotransferase; ALT, alanine aminotransferase; GGT, gamma-glutamyl transferase.

**Table 6:** The sensibility/specificity balance of laboratory parameters in predicting pneumonia in patients with SARS CoV-2.

Parameter	AUC	Standard Error	*p*	95%CI	
				−95%CI	+95%CI
**Blood**					
White blood cells	0.594	0.061	0.150	0.475	0.714
PMN	0.676	0.057	0.007	0.566	0.787
CRP	0.779	0.054	0.001	0.672	0.886
ESR	0.590	0.071	0.170	0.452	0.729
Fibrinogen	0.734	0.066	0.001	0.605	0.863
Ferritin	0.767	0.056	0.001	0.657	0.877
ALT	0.706	0.056	0.002	0.595	0.817
AST	0.769	0.052	0.001	0.668	0.871
GGT	0.684	0.066	0.005	0.554	0.814
Bilirubin	0.758	0.047	0.001	0.665	0.851
Glycemia	0.625	0.061	0.056	0.505	0.746
Creatinine	0.625	0.061	0.057	0.505	0.745

Notes: The test result variable(s): White blood cells, PMN, CRP, ESR, Fibrinogen, Ferritin, ALT, AST, GGT, Bilirubin, Glycemia, and Creatinine have at least one tie between the positive actual state group and the negative actual state group. Statistics may be biased. a Under the nonparametric assumption; b Null hypothesis: true area = 0.5. Abbreviations: AUC, area under the curve; CI, confidence interval; PMN, polymorphonuclear cells; CRP, C reactive protein; ESR, erythrocyte sedimentation rate; AST, aspartate aminotransferase; ALT, alanine aminotransferase; GGT, gamma-glutamyl transferase.

**Table 7:** Mean values of biometric parameters according to pneumonia.

Biometric Measurements	Total n = 106	SARS CoV-2 and Pneumonia n = 26	SARS CoV-2 and Non-Pneumonia n = 80	t-Student Test *p*
Systolic blood pressure	121 ± 26	127 ± 14	119 ± 29	0.152
Diastolic blood pressure	76 ± 18	77 ± 17	75 ± 19	0.684
Heart rate	77 ± 18	79 ± 14	77 ± 19	0.586
SpO_2_	95.97 ± 9.59	95.73 ± 2.48	96.05 ± 10.97	0.884
Min SpO_2_	94.71 ± 9.77	93.46 ± 4.04	95.11 ±11.0	0.457

Abbreviations: SARS CoV-2, Severe Acute Respiratory Syndrome-Corona Virus-2; SpO2, oxygen saturation.

**Table 8:** Treatments in SARS CoV-2 patients with pneumonia.

Treatment	Total n = 106	SARS CoV-2 and Pneumonia n = 26	SARS CoV-2 and Non-Pneumonia n = 80	Chi^2^ Test *p*
Remdesivir	2 (1.9%)	1 (3.8%)	1 (1.3%)	0.433
Anticoagulant	30 (28.3%)	9 (34.6%)	21 (26.3%)	0.417
Kaletra (lopinavir 80 mg and ritonavir 20 mg)	20 (18.9%)	12 (46.2%)	7 (10.0%)	0.001
Dexamethasone	24 (22.6%)	15 (57.7%)	9 (11.3%)	0.001
Antibiotic	37 (34.9%)	20 (76.9%)	17 (21.3%)	0.001
Ceftriaxone	27 (25.5%)	14 (53.8%)	13 (16.3%)	0.001
Augmentin	4 (3.8%)	1 (3.8%)	3 (3.8%)	0.982
Clarithromycin	8 (7.5%)	5 (19.2%)	3 (3.8%)	0.017
Azithromycin	2 (1.9%)	2 (7.7%)	0 (0.0%)	0.012
Paracetamol	84 (79.2%)	21 (80.8%)	63 (78.7%)	0.824
Codeine	17 (16.0%)	5 (19.2%)	12 (15.0%)	0.615
Vitamins	100 (94.3%)	25 (96.2%)	75 (93.8%)	0.631
Antihistamines	6 (5.7%)	2 (7.7%)	4 (5.0%)	0.617

Abbreviations: SARS CoV-2, Severe Acute Respiratory Syndrome-Corona Virus-2.

**Table 9:** Signs and symptoms in relation to unfavorable evolution in patients with SARS CoV-2 and pneumonia.

Sign	AUC	Standard Error	*p*	95%CI	
				−95%CI	+95%CI
Dry cough	0.643	0.077	0.057	0.491	0.795
Productive cough	0.532	0.077	0.668	0.381	0.683
Dyspnoea	0.505	0.075	0.946	0.357	0.653
Asthenia	0.482	0.073	0.814	0.338	0.626
Myalgia	0.605	0.080	0.162	0.448	0.761
Fever	0.571	0.079	0.342	0.416	0.726
Shiver	0.460	0.071	0.596	0.321	0.600
Ageusia	0.504	0.075	0.953	0.357	0.652
Anosmia	0.493	0.074	0.926	0.347	0.639
Oropharyngeal pain	0.516	0.076	0.827	0.367	0.666
Chest pain	0.532	0.077	0.668	0.381	0.683
Paraesthesia	0.528	0.077	0.711	0.376	0.680

Notes: The test result variable(s): Sex, Age, Area, Dry cough, Productive cough, Dyspnoea, Asthenia, Myalgia, Fever, Shiver, Ageusia, Anosmia, Oropharyngeal pain, Chest pain, and Paresthesia have at least one tie between the positive actual state group and the negative actual state group. Statistics may be biased. a Under the nonparametric assumption. B Null hypothesis: true area = 0.5. Abbreviations: AUC, area under the curve; CI, confidence interval.

**Table 10:** IADL evaluation in SARS CoV-2 patients with pneumonia before and after the rehabilitation program.

IADL	Before Rehabilitation Program		Chi^2^ Test	After Rehabilitation Program	Chi^2^ Test
Less Able To	Group I n = 26	Group II n = 80	*p*	Group I n = 26	*p*
feed	24 (92.3%)	13 (16.3%)	0.001	12 (46.2%)	0.001
bath	24 (92.3%)	5 (6.3%)	0.001	13 (50.0%)	0.001
dress	25 (92.2%)	5 (6.3%)	0.001	13 (50.0%)	0.001
walk	25 (96.2%)	1 (1.3%)	0.001	15 (57.7%)	0.001
take medications	13 (50.0%)	25 (31.3%)	0.085	5 (19.2%)	0.021
cook	25 (92.2%)	16 (20.0%)	0.001	18 (69.2%)	0.028
telephone	8 (30.8%)	11 (13.7%)	0.077	5 (19.2%)	0.341
manage finances	17 (65.4%)	11 (13.8%)	0.001	16 (61.5%)	0.775

Abbreviations: IADL, Instrumental Activities of Daily Living.

## Data Availability

Not applicable.
